# Supplementary material for: A Long-Term Comparative Analysis of Endovascular Coiling and Clipping for Ruptured Cerebral Aneurysms: An Individual Patient-Level Meta-Analysis Assessing Rerupture Rates
Source: J Clin Med. 2024 Mar 20;13(6):1778. doi: 10.3390/jcm13061778 (PMC10970744; doi:10.3390/jcm13061778)
Supplement: Supplementary file 1 [file jcm-13-01778-s001.zip › jcm-2915454-supplementary.pdf]

Figure S1: PRISMA checklist

PRISMA 2020 Checklist

Section and Topic	Item #	Checklist item	Location where item is reported
TITLE			
Title	1	A long-term Comparative Analysis of Endovascular Coiling and Clipping for Ruptured Cerebral Aneurysms: An Individual Patient-Level Meta-Analysis Assessing Re-Rupture Rates	Title
ABSTRACT			
Abstract	2	Rerupture after aneurysmatic subarachnoid hemorrhage (aSAH) is rare. However, long-term data of ISAT are limited by high proportions of good grade aSAH patients and small aneurysms. We aim to investigate the impact of Coiling and Clipping regarding the rerupture of the initially ruptured target aneurysms via a literature search in PubMED, Medline, Cochrane, and Embase until 1st August 2023. After the available studies following inclusion and exclusion criteria were screened, the main outcome measures were strictly extracted.	Abstract
INTRODUCTION			
Rationale	3	Aneurysmal subarachnoid hemorrhage (aSAH) is a severe condition with high morbidity and mortality despite recent treatment advances [1]. Complete obliteration of the ruptured aneurysm dome is the preferred therapy [2]. The 2023 AHA/ASA Guidelines suggest clipping or coiling when clinically feasible [3]. In the acute phase and mid-term follow-up, both procedures yield similar results when properly indicated [2, 4]. However, the long-term complication of re-rupture with a second hemorrhage event must be prevented at all costs [5]. A possible cause is an aneurysm remnant that grows over time, leading to a second aSAH [6]. Long-term follow-up is crucial to assess re-rupture rates and compare occlusion methods accurately. Individual patient data (IPD) meta-analysis is the most suitable method [7], but it hasn't been done for re-bleeding rates to date.	Introduction
Objectives	4	Our study aims to conduct an IPD meta-analysis on re-bleeding rates in aSAH patients who underwent surgical clipping or endovascular coiling and were appropriately followed up [7].	Introduction
METHODS			
Eligibility criteria	5	We searched for "aneurysmal subarachnoid hemorrhage rerupture" in Pubmed, Medline, Cochrane and Embase database until 1st August 2023 and found 240 eligible studies. Subsequently, we screened meta-analyses on subarachnoid hemorrhage for further possibly eligible studies. Included were all studies describing rerupture-free survival data of the initially treated ruptured aneurysms ("target aneurysm") after Coiling or Clipping with Kaplan-Meier charts and corresponding patient number at risk tables. We excluded case studies, study protocols, non-clinical trials and meta-analyses. The rerupture event is limited to rebleedings from an already previously ruptured aneurysm which has been successfully treated by coiling or clipping. No data on rerupture form untreated aneurysms at other sites or de novo aneurysms that caused a second aSAH were included.<br>Two reviewers (JW, MV) independently screened abstracts, and full-text articles for two rounds, with any residual conflicts resolved by a third reviewer (EG).	Methods
Information sources	6	We searched for "aneurysmal subarachnoid hemorrhage rerupture" in Pubmed, Medline, Cochrane and Embase database until 1st August 2023 and found 240 eligible studies.	Methods
Search strategy	7	Included were all studies describing rerupture-free survival data of the initially treated ruptured aneurysms ("target aneurysm") after Coiling or Clipping with Kaplan-Meier charts and corresponding patient number at risk tables. We excluded case studies, study protocols, non-clinical trials and meta-analyses. The rerupture event is limited to rebleedings from an already previously ruptured aneurysm which has been successfully treated by coiling or clipping.	Methods
Selection process	8	We performed title screening, abstract screening and in case of uncertainty a whole text screening to search for the eligible studies.	Methods

PRISMA 2020 Checklist

Section and Topic	Item #	Checklist item	Location where item is reported
Data collection process	9	The data collection was performed by two authors independently (MV, JW). First the study titles were screened, then the corresponding abstract and in case of further uncertainty the full-text was screened by two authors (JW and EG) until all retrieved studies were either included or excluded. Two authors (JW, MV) independently extracted the following data from the studies: clinical and imaging characteristics of aSAH patients, prevalence of coiled or clipped ruptured cerebral aneurysms, and data regarding rerupture-free survival in cases with coiled or clipped ruptured cerebral aneurysms. The IPD information of rerupture-free survival was extracted from the published Kaplan-Meier survival charts and patient number at risk tables using Digitizelt (Version 2.5.10 for macOS, Braunschweig, Germany) [10]. This method was performed for each individual subgroup of aSAH patients who underwent coiling or clipping. The extracted rerupture-free survival data and the published number at risk tables were used to reconstruct the Kaplan-Meier curves for each included study using the method of Liu et al. with the R package *IPDfromKM* in R studio (Rstudio, Boston, MA, USA) [11]. Furthermore, the number at risk tables were also created. Afterwards, the reconstructed curves, number at risk tables, estimated HRs, and estimated 95% confidence intervals were compared with those in the original publications. In case of apparent discrepancies, the extraction process was repeated.	Methods
Data items	10a	We analyzed the studies to conduct a meta-analysis according to the following outcomes:<br>1) In case of coiling or clipping for aSAH, following outcomes were analyzed: Probabilities of rerupture of the initially ruptured and treated "target" aneurysm	Methods
Study risk of bias assessment	11	The National Institutes of Health Quality Assessment Tool for observational cohort and cross-sectional studies (NIH-QAT) was used for the assessment of quality and risk of bias of included studies [9].	Methods
Effect measures	12	We measured and reported outcomes in Forest-plots, providing heterogeneity and inconsistency analysis, pooled hazard ratios and statistical significance. Furthermore, a pooled Kaplan-meier chart of the reconstructed IPD was generated.	Methods
Synthesis methods	13a	To see all the eligible studies and reported outcomes, see Table 1	Methods
	13b	To see all the eligible studies and reported outcomes, see Table 1	Methods
	13c	For visualization of our meta-analysis, Forest Plots were created with Review Manager Web (RevMan Web Version 5.4.1 from the Cochrane Collaboration, available at revman.cochrane.org) were presented as figures to selected outcomes. IPD was generated using the R package *IPDfromKM* in R studio (Rstudio, Boston, MA, USA) [11]. Kaplan-Meier curves of rerupture-free survival were constructed for the whole included patient cohort using the R package *Survminer* and *Survival* in R software version 4.3.1 (R Foundation for Statistical Computing, Vienna, Austria)	Methods
	13d	Patient demographics and disease-specific characteristics of the included studies were recorded and compared using the two-sided Pearson´s chi-squared test. The IPD information of all time-to-rerupture data from all the included trials was pooled, and Kaplan-Meier curves of rerupture-free survival were constructed for the whole included patient cohort using the R package *Survminer* and *Survival* in R software version 4.3.1 (R Foundation for Statistical Computing, Vienna, Austria). The 1-, 6-, and 10-years rerupture-free survival probabilities were calculated. The hazard ratios (HR) of each individual study as well as the pooled HR and corresponding 95% confidence intervals (CI) between clipped and coiled aSAH patients were estimated.<br>In the two-stage meta-analysis, we combined the estimated hazard ratios (HRs) and their corresponding 95% CI from individual studies using a random-effects model, employing the generic inverse variance method. The calculated HRs were logarithmically transformed (LN). For each study, the standard error (SE) was derived from the 95% CI using the formula: SE = (LN(upper CI limit) – LN(lower CI limit)) / 3.92, as outlined in the Cochrane Handbook for Systematic Reviews of Interventions, Version 6.4 [12]. Heterogeneity across the included studies was	Methods

PRISMA 2020 Checklist

Section and Topic	Item #	Checklist item	Location where item is reported
		evaluated using I^2 statistics, with a threshold of >50% displaying substantial heterogeneity [13]. The aggregated results were presented in forest plots utilizing Review Manager Web (RevMan Web Version 5.4.1 from The Cochrane Collaboration). Weight of the relative contribution of the included studies, based on the sample size, was considered concerning the estimation of the treatment effects. A significance level of $p < 0.05$ was considered statistically significant. To evaluate publication bias, we performed a two-step approach. Firstly, visually assessment via funnel plots was performed. Funnel plots were created using the R package *metafor*. Secondly, Begg´s test was performed to statistically evaluate the asymmetry of the data [14].	
	13e	Not available	Methods
	13f	Not available	Methods
Reporting bias assessment	14	According to our strict inclusion criteria and high quality of included studies, we do not suppose to have bias due to missing results. Risk of bias assessment was performed for all included studies	Methods
Certainty assessment	15	Quality assessment was performed and the risk of bias assessment is summarized in supplemental figure 2.	Methods
RESULTS			
Study selection	16a	Process of the search and selection is summarized in Figure 2.	Figure 1
	16b	Excluded studies are summarized in supplementary Figure 2.	Figure 1
Study characteristics	17	Included studies are summarized in table 1.	Table 1
Risk of bias in studies	18	Risk of bias analysis is reported in supplementary figure 2.	Supplemental Figure 2
Results of individual studies	19	Analysis of each outcome is separately reported on with it´s own forest plot in Figure 3.	Figures 3
Results of syntheses	20a	Risk of bias assessment is summarized in the supplementary figure 2. Figure 4 represents the funnel plot visualizing the publication bias	Suppl. Figure 2 & Figure 4
	20b	Results of the statistical syntheses are summarized in the Figures 2, and Figure 3.	Figures 2 & 3
	20c	Results regarding heterogeneity are presented in the Figure 3.	Figure 3
	20d	Presented	Results
Reporting biases	21	Risk of bias assessment is shown in Figure 4	Figure 4
Certainty of evidence	22	Not available	NA
DISCUSSION			
Discussion	23a	Provide a general interpretation of the results in the context of other evidence.<br>Presented	Discussion

PRISMA 2020 Checklist

Section and Topic	Item #	Checklist item	Location where item is reported
	23b	Discuss any limitations of the evidence included in the review.<br>Presented	Discussion, last paragraph
	23c	Discuss any limitations of the review processes used.<br>Presented	Discussion, last paragraph
	23d	Discuss implications of the results for practice, policy, and future research.<br>Presented	Discussion
OTHER INFORMATION			
Registration and protocol	24a	The study was registered in the "International Prospective Register of Systematic Reviews" (PROSPERO) in 2023 (CRD42023463253)	Methods
	24b	The study was registered in the "International Prospective Register of Systematic Reviews" (PROSPERO) in 2023 (CRD42023463253)	Methods
	24c	No amendments to describe	
Support	25	There is no financial conflict of interest to declare	NA
Competing interests	26	No competing interests of the authors to delcare	NA
Availability of data, code and other materials	27	Template data collection form, data extracted and used for the analysis are reported in the section "methods". The softwares Revman, R packages (IPDfromKM, SURVMINER, METAFOR, and SURVIVAL are available online	Methods

From: Page MJ, McKenzie JE, Bossuyt PM, Boutron I, Hoffmann TC, Mulrow CD, et al. The PRISMA 2020 statement: an updated guideline for reporting systematic reviews. BMJ 2021;372:n71. doi: 10.1136/bmj.n71

For more information, visit: http://www.prisma-statement.org/

Figure S2: NIH-Quality assessment of the included studies

NIH Quality assessment **Controlled Intervention Studies**	**Was the study described as randomized, a randomized trial, a randomized clinical trial, or an RCT?**	**Was the method of randomization adequate (i.e., use of randomly generated assignment)?**	**Was the treatment allocation concealed (so that assignments could not be predicted)?**	**Were study participants and providers blinded to treatment group assignment?**	**Were the people assessing the outcomes blinded to the participant's group assignments?**	**Were the groups similar at baseline on important characteristics that could affect outcomes (e.g., demographics, risk factors, co-morbid conditions)?**	**Was the overall drop-out rate from the study at endpoint 20% or lower of the number allocated to treatment?**	**Was the differential drop-out rate (between treatment groups) at endpoint 15 percentage points or lower?**	**Was there high adherence to the intervention protocols for each treatment group?**	**Were other interventions avoided or similar in the groups (e.g., similar background treatments)?**	**Were outcomes assessed using valid and reliable measures, implemented consistently across all study participants?**	**Did the authors report that the sample size was sufficiently large to be able to detect a difference in the main outcome between groups with at least 80% power?**	**Were outcomes reported or subgroups analyzed prespecified (i.e., identified before analyses were conducted)?**	**Were all randomized participants analyzed in the group to which they were originally assigned, i.e., did they use an intention-to-treat analysis?**
**Molyneux 2009**	**Y**	**Y**	**Y**	**N**	**N**	**Y**	**Y**	**Y**	**Y**	**NA**	**Y**	**Y**	**Y**	**Y**

Y = Yes; N = No; NA = Not applicable

NIH Quality assessment **Observational cohort and Cross-sectional** studies	**Research question or objective stated**	**Study population defined**	**Participant rate at least 50% from eligible**	**Subject recruitment and eligibility criteria**	**Sample size justification**	**Exposure(s) measured prior to the outcome(s)**	**Sufficient time frame**	**Examination of different levels of exposure as related to the outcome**	**Definition and validation of the exposure measures**	**Exposure(s) assessed more than once**	**Definition of outcome measures**	**Blinded assessors**	**Loss to follow-up after baseline of 20% or less**	**Statistical measure and adjustment of key confounding variables**
**CARAT 2006**	**Y**	**Y**	**Y**	**Y**	**N**	**Y**	**Y**	**NA**	**Y**	**N**	**Y**	**N**	**NA**	**N**

Y = Yes; N = No; NA = Not applicable
